# Risk profiles of the preterm behavioral phenotype in children aged 3 to 18 years

**DOI:** 10.3389/fped.2023.1084970

**Published:** 2023-10-19

**Authors:** Grace C. Fitzallen, Alison Griffin, H. Gerry Taylor, James N. Kirby, Helen G. Liley, Samudragupta Bora

**Affiliations:** ^1^ School of Psychological Sciences, College of Health and Medicine, University of Tasmania, Launceston, TAS, Australia; ^2^ Mater Research Institute, Faculty of Medicine, The University of Queensland, Brisbane, QLD, Australia; ^3^ School of Psychology, Faculty of Health and Behavioural Sciences, The University of Queensland, Brisbane, QLD, Australia; ^4^ Statistics Unit, QIMR Berghofer Medical Research Institute, Herston, QLD, Australia; ^5^ Center for Biobehavioral Health, Abigail Wexner Research Institute at Nationwide Children's Hospital, and Department of Pediatrics, The Ohio State University, Columbus, OH, United States; ^6^ Department of Pediatrics, University Hospitals Rainbow Babies & Children's Hospital, Case Western Reserve University School of Medicine, Cleveland, OH, United States

**Keywords:** anxiety, attention-deficit/hyperactivity disorder, autism spectrum disorder, behavior, mental health, neurodevelopment, preterm birth, psychopathology

## Abstract

**Objective:**

Characterize the Preterm Behavioral Phenotype in children born preterm by identifying distinct profiles based on patterns of symptomatology or severity of the risk for attention-deficit/hyperactivity disorder, autism spectrum disorder, and anxiety, and determine their associations with child sex, gestational age, and chronological age.

**Methods:**

Sample comprised 2,406 children born preterm aged 3–18 years with primary caregiver behavioral ratings on the standardized Strengths and Weaknesses of ADHD Symptoms and Normal Behavior Scale, Social Responsiveness Scale, and Preschool Anxiety Scale or Screen for Child Anxiety and Related Emotional Disorders.

**Results:**

Statistical fit indices of latent profile analysis supported a 3-profile model as optimal. Using this model, 75% of children born preterm were identified as having low expression, 20% moderate expression, and 5% high expression profiles of the Preterm Behavioral Phenotype described as co-occurring symptomatology of attention-deficit/hyperactivity disorder, autism spectrum disorder, and anxiety. Male children were more likely than females to be categorized in the moderate expression [Relative Risk Ratio (RRR) = 1.29, 95% CI = 1.05–1.59], and high expression profiles (RRR = 1.77, 95% CI = 1.17–2.66). Children born extremely preterm were more likely than those born moderate/late preterm to be categorized in the moderate expression (RRR = 1.68, 95% CI = 1.30–2.19) and high expression profiles (RRR = 2.06, 95% CI = 1.31–3.25). Finally, those in the school-age (RRR = 1.68, 95% CI = 1.32–2.14; RRR = 1.95, 95% CI = 1.21–3.13), early adolescence (RRR = 1.85, 95% CI = 1.38–2.48; RRR = 2.61, 95% CI = 1.53–4.44) and late adolescence (RRR = 2.09, 95% CI = 1.38–3.19; RRR = 2.28, 95% CI = 1.02–5.08) periods were more likely than those in the preschool period to be categorized in the moderate and high expression profiles, respectively.

**Conclusion:**

A quarter of children born preterm were at elevated risk for manifesting symptomatology across all three domains of the Preterm Behavioral Phenotype. Findings emphasize accounting for symptom co-occurrence of this phenotype in neurodevelopmental follow-up and psychosocial interventions to optimize child outcomes.

## Introduction

Children born preterm are 2–3 times more likely to be diagnosed with attention-deficit/hyperactivity disorder (ADHD; inattention, hyperactivity/impulsivity), autism spectrum disorder (ASD; socialization difficulties and restrictive interests and/or behaviors), and anxiety compared with term-born peers (1–3). These domains are consistently identified as the most common behavioral and socioemotional difficulties in this population (4–7). Consequently, the Preterm Behavioral Phenotype was proposed (8), describing distinct co-occurrence of ADHD, ASD, and anxiety symptomatology, with this manifestation pattern not commonly seen in term-born children (8, 9). For example, children born preterm at risk of ADHD are more likely to experience inattentive subtype symptomatology and less likely comorbid oppositional/conduct difficulties (8). ASD is likely to manifest through selective difficulties compared with broader pervasive difficulties (10–13), and there are higher rates for internalizing difficulties than anxiety diagnoses (6). Of note, children born preterm typically present with high-prevalence and low-severity difficulties falling short of clinical diagnoses, making detection challenging (14).

Concerningly, more than 10 years after proposal, there remains insufficient research describing the frequency and predictive factors of the Preterm Behavioral Phenotype. The most recent review (9) identified only three studies explicitly investigating the Preterm Behavioral Phenotype using data profile analysis approaches. Using latent class analysis, a person-centered approach identifying similar patterns of responses from observable categorical measures (15, 16), the study by Johnson et al. (17) identified three classes of behavioral difficulties in 2-year-old children born moderate/late preterm (MLPT; *n *= 638). In contrast, two classes were identified in the term-born group. The third class, only identified within the MLPT group, most closely aligns with the Preterm Behavioral Phenotype, encompassing elevated symptomatology for poorer socioemotional competency, cognitive impairment, ASD behaviors, and delayed language development, relative to the other two classes. The greatest proportion of the MLPT sample was in Class 1 with outcomes determined “optimal” within the typical range (67%), followed by Class 2 with suboptimal outcomes (26%), and Class 3 with elevated difficulties (7%). Class 3 profile classification was associated with male sex (odds ratio [OR]: 5.36, 95% confidence interval [CI]: 1.90–15.12) and preeclampsia (OR: 3.67, 95% CI: 1.58–8.51). A study limitation is the restricted statistical power of the sample size to detect differences by the degree of prematurity; however, greater gestational age (OR: 1.57, 95% CI: 1.02–2.40) was associated with elevated difficulties.

Using latent profile analysis (LPA), a continuous variable equivalent to latent class analysis (15, 16), the study by Burnett et al. (18) identified four profiles of behavioral difficulties in 8-year-old children born extremely preterm (EPT)/extremely low birthweight (*n *= 181) and term-born children (*n *= 185). Preterm and term-born children were present in all profiles, so unlike the study by Johnson et al. (17), there were no profiles unique to the preterm group. The greatest proportion of children born preterm was in Profile 1 with minimal difficulties (55%), followed by Profile 2 with a subclinical elevation of difficulties (20%), Profile 3 with greater elevation in emotional, behavioral, and conduct difficulties (no elevation in peer difficulties; 16%), and Profile 4 with a substantial elevation of difficulties in all areas (8%). Severe behavioral difficulties were associated with lower intellectual functioning, lower literacy and numeracy scores, and EPT birth.

The study by Lean et al. (19) also using LPA identified four profiles of parent- and teacher-reported psychiatric and neurodevelopmental impairments in 5-year-old children born very preterm (VPT; *n *= 85) and at-term (*n *= 40). As with Burnett et al. (18), VPT and term-born children were present in all profiles. The greatest proportion of VPT-born children were profiled in the at-risk group (45%) with typical neurodevelopment and slightly elevated psychiatric ratings within the typical range; followed by the typically-developing group (27%) with all scores within the typical range; the psychiatric group (13%) with mild-moderate internalizing and moderate-severe ADHD, ASD, externalizing symptomatology and executive functioning impairment; and the school-based inattentive/hyperactive group (15%) with mild-moderate internalizing/externalizing and moderate-severe ADHD and ASD.

Taken together, these studies (17–19) collectively indicate that the phenotype has categorically distinct groupings of specific, co-occurring symptomatology. Nonetheless, it remains unclear if there are different subprofiles of the Preterm Behavioral Phenotype, or a single profile varying by levels of severity. Further, each study focused on children of specific ages and preterm birth groups, relying on broad domain measures (i.e., internalizing behavior rather than anxiety symptomatology), limiting the external validity of the findings.

To address these limitations and discrepancies in previous research, we collected a large dataset of caregiver-reported behavioral and socioemotional outcomes on validated instruments in a cross-sectional study including preterm-born children of multiple ages (3–18 years) and degrees of prematurity. We aimed to characterize the Preterm Behavioral Phenotype and determine whether there are distinct profiles of symptomatology (based on patterns of symptomatology or severity of risk) by undertaking LPA using domain and subdomain outcomes of ADHD, ASD, and anxiety. Finally, we aimed to evaluate the role of caregiver-reported child characteristics (i.e., sex, gestational age, chronological age) as risk factors for each profile.

## Methods

### Sample

Primary caregivers of children born preterm (<37 weeks gestation) with English as their primary language (not necessarily native) and residing in Australia, Canada, New Zealand, United Kingdom/Ireland, or United States of America were recruited through parent support organizations between October 2019 and February 2020 to report on child behavioral outcomes. A secure web-based portal was used to administer screening and outcome questionnaires. Caregivers with >1 preterm-born child (i.e., multiple birth or more than one preterm birth) were asked to refer to their youngest child born preterm. Caregivers were eligible if this preterm-born child did not have a chromosomal anomaly, fetal alcohol spectrum disorder, and/or developmental disability (IQ<70), as determined by caregiver-report of previous diagnosis or testing.

Furthermore, the caregiver report of the child's gestational age needed to include responses “*confident*”, “*very confident*”, or “*extremely confident*” on a 5-point Likert scale. To further ensure the accuracy of gestational age as the primary inclusion criterion, respondents were asked to specify it at both pre- and post-consent using two different response formats. Of responders (*n *= 2,623/3,328 eligible; 79%), 97% were congruent in gestational age reporting. The remaining 3% (*n *= 80) were excluded from this study. Respondents with partial data were also excluded (*n *= 137/2,623; 5%). Consequently, the final sample comprised 2,406 children born preterm with a 72% participation rate. Analysis of those excluded and included showed that excluded children were less likely to have received oxygen therapy at 36 weeks (*p *= .003) and more likely to be older at the time of assessment (*p *= .002).

This study was designed and conducted in accordance with the American Association for Public Opinion Research best practice for survey research. The study protocol was reviewed and approved by the University of Queensland Health and Behavioural Sciences, Low & Negligible Risk Ethics Committee. Electronically documented informed consent to participate in this study was provided by all the participants.

### Measures

Child outcomes were evaluated based on primary caregiver-reports at a single time-point for three behavioral and socioemotional domains using age-appropriate, standardized screening instruments. ASD was assessed in alignment with the Diagnostic and Statistical Manual of Mental Disorders, Fifth Edition, and ADHD and anxiety with the Diagnostic and Statistical Manual of Mental Disorders, Fourth Edition (DSM-IV). It is noteworthy no conceptual differences exist for ADHD and anxiety between editions. Across all instruments, caregivers were asked to consider their responses about the child's behavior during the past six months.

*ADHD* was assessed using the 18-item Strengths and Weaknesses of ADHD Symptoms and Normal Behavior Scale (SWAN) (20). The instrument includes subdomains of inattention and hyperactivity/impulsivity. For this study, caregivers indicated the extent to which each statement applied to their child on a 4-point Likert scale from “*not at all*” to “*very much*” to represent a disorder-identification approach in line with accompanying assessments (20). The SWAN has strong construct validity to DSM-IV and convergent validity with validated measures of ADHD including clinician diagnosis (20, 21).

*ASD* was assessed using the 65-item Social Responsiveness Scale, Second Edition (22). This instrument encompasses five subdomains: social awareness, social cognition, social motivation, social communication, and restrictive/repetitive behavior. Caregivers indicated the extent to which each statement best described their child's behavior on a 4-point Likert scale from “*not true*” to “*almost always true*”. While this scale is screening in nature, it has been shown to consistently demonstrate strong construct validity and predictive validity, including the accurate identification of those with and without ASD 92% of the time (23).

*Anxiety* was assessed using two standardized instruments dependent on the child's age at the time of assessment: The Preschool Anxiety Scale (PAS) (24) and the Screen for Child Anxiety and Related Emotional Disorders (SCARED) (25). The PAS is a 34-item instrument used to evaluate anxiety in children aged 3–7 years across five subdomains (generalized anxiety, social anxiety, separation anxiety, obsessive-compulsive symptomatology, and physical injury fears). Caregivers indicated their agreement on a 5-point Likert scale from “*not true at all*” to “*very often true*”. The PAS has exceptional construct validity to DSM-IV diagnoses (24, 26), and convergent validity with other established anxiety measures for young children (27).

The SCARED is a 41-item instrument screening for anxiety in children aged 8–18 years across five subdomains (generalized anxiety, social anxiety, separation anxiety, panic and somatic symptomatology, and school avoidance). Caregivers indicated item agreement on a 3-point Likert scale from “*not true or hardly ever true*” to “*very true or often true*”. The SCARED consistently demonstrates strong construct validity to DSM-IV (28), and convergent validity with established evaluations of anxiety including clinician-rated measures (29, 30).

The anxiety total score was calculated using all developmentally appropriate subdomains listed above. To ensure consistency for subdomain analyses, only compatible subdomains of generalized, social, and separation anxiety were used.

### Statistical analyses

Sample characteristics were described using percentage (numerator/denominator) for categorical variables and mean with standard deviation (SD) for continuous variables. LPA was performed using STATA, v15.1 (31). All domain and subdomain outcome raw scores were transformed into *z*-scores with a mean of 0 and SD of 1. To select the optimal number of profiles for LPA, we examined model fit indices Akaike Information Criterion (AIC), Bayesian Information Criterion (BIC), Sample Size Adjusted Bayesian Information Criterion (SABIC), Lo-Mendell-Rubin-Adjusted Likelihood Ratio Test (LMRALRT), Vuong-Lo-Mendell-Rubin Likelihood Ratio Test (VLMRLRT), and Entropy statistics. Entropy values range from 0 to 1, with higher values indicating better delineation between profiles. Where fit statistics are inconsistent on the optimal number of profiles, consideration is given to entropy values, size of the smallest profile, interpretability, and theoretical considerations. Model specification allowing for covariance in error terms was used due to significant residual correlation between outcomes after profile assignment. After the selection of the optimal number of profiles, LPA was performed for domain outcomes using total scores (three outcomes), and subdomain outcomes using subscale scores (10 outcomes). Multinomial logistic regression was performed to determine the relative risk ratio (RRR) for being categorized in Profile 2 and Profile 3, relative to Profile 1 by child sex (male, female), child gestational age (EPT, VPT, MLPT), and chronological age groups (preschool 3–5, school-age 6–9, early adolescence 10–14, late adolescence 15–18 years).

## Results

The characteristics of the final sample can be seen in Table 1. Across the four age groups, the largest proportion of children were in the preschool age group (47%) followed by school-age (31%), early adolescence (16%), and late adolescence (6%). Slightly more than half the sample was male, and compared to the typical gestation profile of preterm births, infants born extremely preterm were slightly over-represented (24% of the sample).

**Table 1:** Characteristics of the sample.

Characteristics, % (Numerator/Denominator)	*N *= 2,406
Child neonatal	
Gestational age, mean ± SD, weeks	31 ± 4
Extremely preterm, <28 weeks	23.7 (569/2,406)
Very preterm, 28–<32 weeks	32.6 (785/2,406)
Moderate/late preterm, 32–<37 weeks	43.7 (1,052/2,406)
Birthweight, mean ± SD, grams	1,517 ± 677 [*n *= 2,050]
Male sex	53.2 (1,279/2,406)
Multiple birth	31.6 (631/2,000)
Confirmed neonatal infection	27.7 (538/1,945)
Oxygen therapy at 36 weeks	38.9 (786/2,021)
Severe brain injury or abnormality	6.1 (126/2,057)
Child concurrent	
Chronological age, mean ± SD, years	7 ± 4
Preschool, 3–5 years	46.7 (1,123/2,406)
School-age, 6–9 years	31.1 (748/2,406)
Early adolescence, 10–14 years	16.4 (394/2,406)
Late adolescence, 15–18 years	5.9 (141/2,406)
Developmental diagnosis	
Neurosensory	9.8 (202/2,051)
Neurobehavioral	28.6 (587/2,051)
Physical and chronic condition	13 (267/2,051)
Developmental intervention for >6 months	41.6 (851/2,048)
Behavioral counseling	0.7 (14/2,057)
Mental health intervention	5.3 (110/2,057)
Country of residence	
Australia	36 (866/2,406)
Canada	13 (312/2,406)
New Zealand	18.9 (454/2,406)
United Kingdom/Ireland	15.8 (381/2,406)
United States of America	16.3 (393/2,406)
Maternal at childbirth	
Maternal age, mean ± SD, years	31 ± 5 [*n *= 2,045]
Minority race/ethnicity	8.8 (178/2,030)
Low education [high school graduate or below]	14.4 (347/2,406)
Low family socioeconomic status [unemployed, unskilled, semi-skilled]	20.7 (497/2,406)
Single parent family	6.1 (125/2,044)

### Characterizing the preterm behavioral phenotype

**Figure 1:**
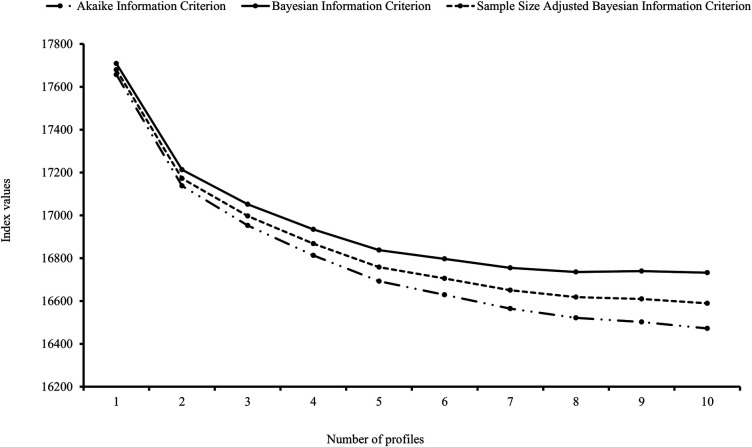
Model fit indices of latent profiles.

*Determining the Optimal Number of Profiles*. Profiles were empirically derived from ADHD, ASD, and anxiety domains. Model fit indices were obtained and examined for 1–10-profile models (see Supplementary Table S1). The AIC, BIC, and SABIC values did not reach a definitive low point across the models, however, the largest decline occurred between 1- and 2-profile models, with smaller decreases between 2- and 5-profile models, and smaller again from the 7-profile model (see Figure 1). Considering LMRALRT and VLMRLRT log-likelihood statistics, there was strong evidence (*p *< .001) to support the 2-profile over the 1-profile model, and 3-profile over the 2-profile model. The 4-profile model was not statistically better than the 3-profile model (*p *> .05). Acceptable entropy values were evident across 2–6-profile models (>.80). Taken together, the 3-profile model was determined to be the most appropriate for this sample and retained for analyses.

**Figure 2:**
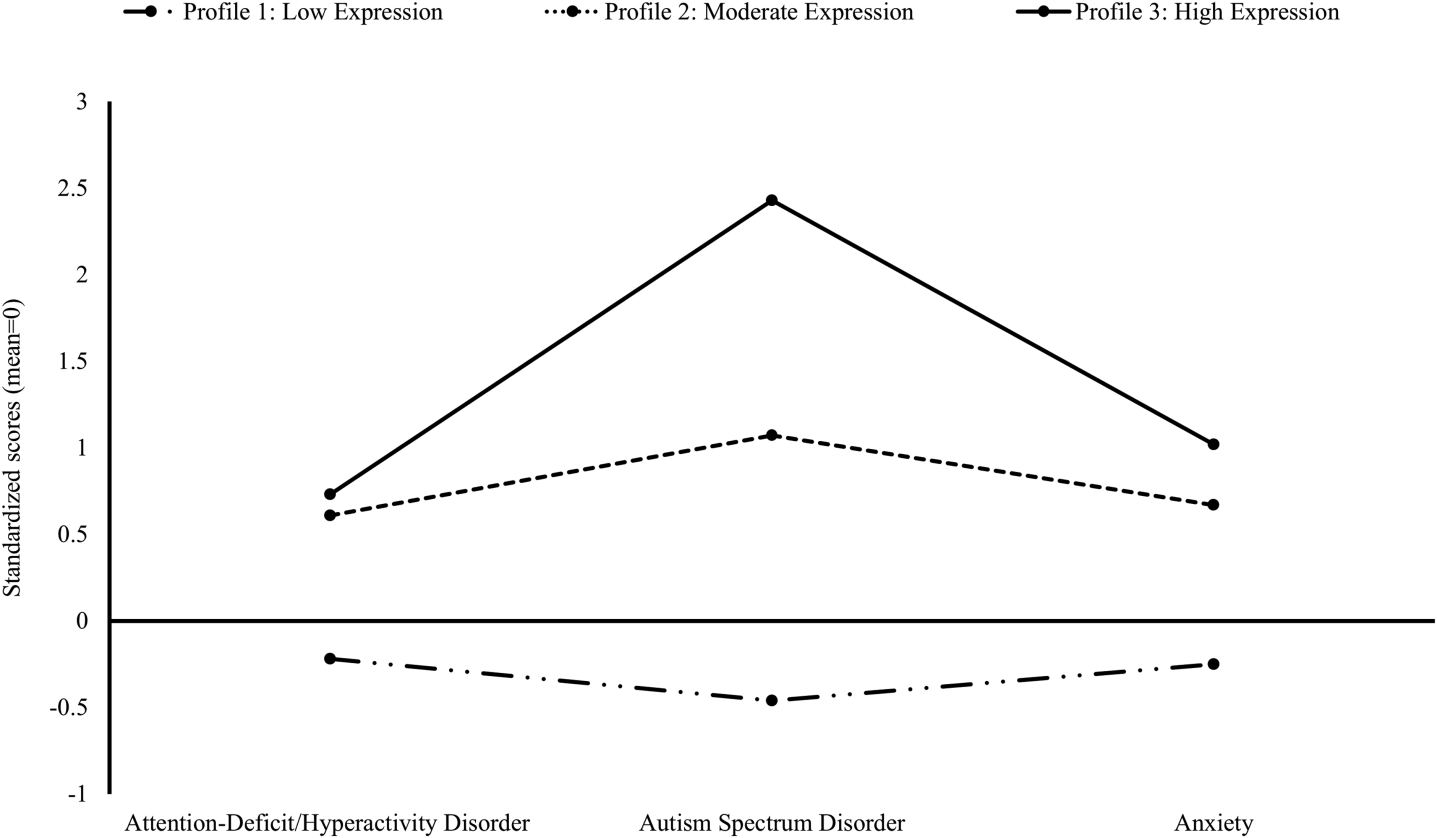
Latent profile analysis for domain scores for ADHD, ASD, and anxiety.

**Figure 3:**
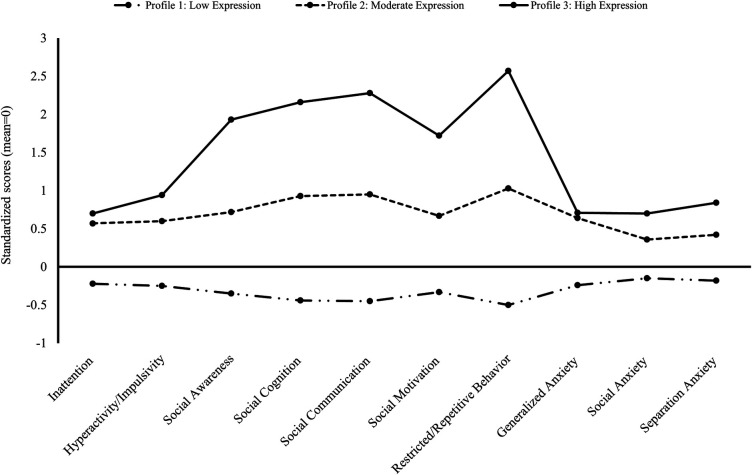
Latent profile analysis for subdomain scores for ADHD, ASD, and anxiety.

*LPA at Domain and Subdomain Levels*. Using the 3-profile model for domain outcomes (see Table 2 and Figure 2), 75% of children born preterm were identified within the low expression profile (95% CI: 72%–78%; “Profile 1: Low Expression”) with scores across all domains within 0.5 SD below the mean of this sample. Further, 20% had a moderate expression profile (95% CI: 18%–23%; “Profile 2: Moderate Expression”), with scores across all domains 0.5–1 SD above the mean. Finally, 5% of children had a high expression profile (95% CI: 4%–7%; “Profile 3: High Expression”), with scores up to 2.5 SD above the mean. The 3-profile model of subdomain outcomes demonstrated a similar pattern (see Table 3 and Figure 3, and Supplementary Figure 1) with 73% having low expression (95% CI: 71%–75%), 21% moderate (95% CI: 19%–24%), and 5% high expression (95% CI: 4%–7%) profiles. A different pattern emerged when comparing moderate and high expression profiles, whereby ASD and associated subdomains differentiated children. Specifically, score differentiation was greater between ASD-related outcomes and ADHD- and anxiety-related outcomes in the high expression profile compared with scores in the moderate expression profile, whereby outcome scores were less differentiated.

**Table 2:** Probability of profile membership and associated scores for domain outcomes.

Characteristics	3-Profile model		
	Profile 1: low expression	Profile 2: moderate expression	Profile 3: high expression
Membership probability (95% confidence interval)	.75 (.72–.78)	.20 (.18–.23)	.05 (.04–.07)
Standardized domain scores, mean (95% confidence interval)			
ADHD	−0.22 (−0.27–−0.17)	0.61 (0.49–0.72)	0.73 (0.52–0.94)
ASD	−0.46 (−0.50–−0.42)	1.07 (0.94–1.20)	2.43 (2.27–2.59)
Anxiety	−0.25 (−0.30–−0.20)	0.67 (0.55–0.79)	1.02 (0.81–1.24)

Standardized subdomain values represent the standard deviation from the mean (value of 0). Negative values represent behavior below the mean for the sample.

**Table 3:** Probability of profile membership and associated scores for subdomain outcomes.

Characteristics	3-Profile model		
	Profile 1: low expression	Profile 2: moderate expression	Profile 3: high expression
Membership probability (95% confidence interval)	.73 (.71–.75)	.21 (.19–.24)	.05 (.04–.07)
Standardized subdomain scores, mean (95% confidence interval)			
ADHD			
Inattention	−0.22 (−0.27–−0.17)	0.57 (0.47–0.67)	0.70 (0.52–0.88)
Hyperactivity/impulsivity	−0.25 (−0.30–−0.20)	0.60 (0.51–0.70)	0.94 (0.77–1.12)
ASD			
Social awareness	−0.35 (−0.39–−0.31)	0.72 (0.63–0.81)	1.93 (1.77–2.09)
Social cognition	−0.44 (−0.47–−0.40)	0.93 (0.84–1.02)	2.16 (2.01–2.30)
Social communication	−0.45 (−0.48–−0.41)	0.95 (0.86–1.04)	2.28 (2.14–2.41)
Social motivation	−0.33 (−0.38–−0.29)	0.67 (0.57–0.77)	1.72 (1.55–1.89)
Restricted/repetitive behavior	−0.50 (−0.53–−0.47)	1.03 (0.96–1.11)	2.57 (2.46–2.68)
Anxiety			
Generalized anxiety	−0.24 (−0.29–−0.19)	0.64 (0.54–0.74)	0.71 (0.52–0.90)
Social anxiety	−0.15 (−0.20–−0.11)	0.36 (0.26–0.47)	0.70 (0.50–0.89)
Separation anxiety	−0.18 (−0.23–−0.14)	0.42 (0.32–0.52)	0.84 (0.64–1.04)

Standardized subdomain values represent the standard deviation from the mean (value of 0). Negative values represent behavior below the mean for the sample.

### Associations between profile membership and child factors

Compared with the low expression referent profile, male children had a significant RRR of 1.29 (95% CI: 1.05–1.59) relative to females to be categorized in the moderate expression profile, and an RRR of 1.77 (95% CI: 1.17–2.66) to be categorized in high expression profile (see Table 4).

**Table 4:** Risk for membership in moderate and high expression relative to low expression profiles.

	Subgroups	Relative risk ratio (95% confidence interval)
Profile 2: moderate expression		
Child sex	Female	Referent
	Male	1.29 (1.05–1.59)*
Gestational age	Moderate/late preterm, 32–<37 weeks	Referent
	Very preterm, 28–<32 weeks	1.25 (0.98–1.60)
	Extremely preterm, <28 weeks	1.68 (1.30–2.19)*
Chronological age	Preschool, 3–5 years	Referent
	School-age, 6–9 years	1.68 (1.32–2.14)*
	Early adolescence, 10–14 years	1.85 (1.38–2.48)*
	Late adolescence, 15–18 years	2.09 (1.38–3.19)*
Profile 3: high expression		
Child sex	Female	Referent
	Male	1.77 (1.17–2.66)*
Gestational age	Moderate/late preterm, 32–<37 weeks	Referent
	Very preterm, 28–<32 weeks	0.84 (0.51–1.39)
	Extremely preterm, <28 weeks	2.06 (1.31–3.25)*
Chronological age	Preschool, 3–5 years	Referent
	School-age, 6–9 years	1.95 (1.21–3.13)*
	Early adolescence, 10–14 years	2.61 (1.53–4.44)*
	Late adolescence, 15–18 years	2.28 (1.02–5.08)*

* *p* < .05.

Each child age group had a significantly greater risk to be categorized in the moderate and high expression profiles compared with the preschool-aged referent group. Compared with the low expression profile, school-aged children (RRR: 1.68, 95% CI: 1.32–2.14), early adolescents (RRR: 1.85, 95% CI: 1.38–2.48), and late adolescents (RRR: 2.09, 95% CI: 1.38–3.19) were significantly more likely than preschool-aged children to be categorized in the moderate expression profile. Further, school-aged children (RRR: 1.95, 95% CI: 1.21–3.13), early adolescents (RRR: 2.61, 95% CI: 1.53–4.44), and late adolescents (RRR: 2.28, 95% CI: 1.02–5.08) were significantly more likely than preschool-aged children to be in the high expression profile.

For gestational age, compared with the low expression profile, children born EPT were significantly more likely than those born MLPT to be categorized in the moderate (RRR: 1.68, 95% CI: 1.30–2.19) and high (RRR: 2.06, 95% CI: 1.31–3.25) expression profiles. The risk was not significantly greater for children born VPT compared with MLPT to be categorized in the moderate (RRR: 1.25, 95% CI: 0.98–1.60) and high (RRR: 0.84, 95% CI: 0.51–1.39) expression profiles.

Independent LPA analyses were conducted for child sex, gestational age, and chronological age subgroups. The probabilities of profile membership for associated subgroups were consistent with the above results (see Supplementary Table S2).

## Discussion

This study characterizes the Preterm Behavioral Phenotype in a relatively large sample of preterm-born children and adolescents. Our research advances the current understanding of this phenotype by including children from a wide age range, across the full spectrum of preterm birth, with profiling analysis performed for both domain and subdomain outcomes. Using LPA of caregiver-reported outcomes on standardized behavioral screening instruments, we identified promising findings with most children in the low expression profile, meaning that study instruments identified few or no difficulties for the behavioral and socioemotional difficulties of interest. We found a quarter of children born preterm demonstrated moderate or high expression profiles, with consistent findings at domain and subdomain levels. Consistent with previous research (17–19), only a small proportion of children born preterm (<5%) were categorized in the high expression profile, with a notable proportion of children expressing low to moderate difficulties. Memberships in the moderate and high expression profiles (compared with the low expression profile) were associated with male sex, earlier gestational age, and older chronological age. Previous studies of behavioral and socioemotional outcomes in this population have reported similar associations (32). While all subgroups of prematurity and child age had greater relative risk compared with referent groups, a much larger study with longitudinal data is needed to statistically define these relationships. Specifically, the relationship with chronological age may be affected by observer bias because behavioral difficulties typically become more apparent with increasing age and school-related transitions.

When evaluating patterns of symptomatology across the profiles, there was a similar pattern of co-occurrence for low and moderate expression profiles, differentiated only by severity; however, a different pattern emerged for the high expression profile, whereby higher screening scores for ASD were more common. This was evident across all five ASD subdomains, with the highest scores for restricted/repetitive behavior. These findings indicate that ASD and associated difficulties may be a key discriminator between children with moderate and high expression difficulties. Therefore, screening for these behaviors is particularly important for detecting children who may manifest the co-occurrence seen in the Preterm Behavioral Phenotype.

It needs to be acknowledged that varying measurement properties of instruments used in this study to characterize the Preterm Behavioral Phenotype may be associated with the data-driven LPA approach. To avoid similar challenges in future research and clinical practice, it is recommended that a specific Preterm Behavioral Phenotype screening instrument is developed to assist in the timely and cost-effective detection of children most at risk for co-occurring symptomatology.

Limitations of this study include the exclusion of children with severe developmental disabilities. Approximately 70% of children diagnosed with ASD have lower intellectual functioning compared with those not diagnosed with ASD (33). Therefore, our exclusion of these children may have led to an underestimation of risk and restricted our investigation of intellectual impairment as a potential discriminant for severity. While this study was able to recruit a large sample of caregivers through parent support organizations which may not have been achievable through other recruitment avenues (e.g., neonatal follow-up clinics), this may have resulted in a nonrepresentative sample. Those recruited may have different characteristics from those not engaged with these social support groups. Further, while standardized, well-validated instruments used in epidemiological research and as components of multifaceted screening batteries were selected for this study, they do not equate to diagnostic assessment. Discordance has been reported between caregiver- and adolescent-report on behavioral outcomes, with parents typically reporting greater rates of difficulties (6). This may have led to the overestimation of symptom expression. Furthermore, while this study was able to investigate the relative risk associated with child sex, gestational age, and chronological age, investigation of early neonatal risk factors, biomarkers, other developmental difficulties, and longitudinal developmental trajectory of profile manifestation was not possible. Finally, this study did not include a term-born comparison group.

In conclusion, we found that a quarter of our sample born preterm had a moderate or high expression profile for co-occurring ADHD, ASD, and anxiety symptomatology, previously described as the Preterm Behavioral Phenotype. We found that this pattern of symptomatology was associated with male sex, earlier gestational age, and older child age. Findings highlight the need to account for symptom co-occurrence of this phenotype in neurodevelopmental follow-up and psychosocial interventions to optimize child outcomes. With consideration of the co-occurring, low to moderate expression of these difficulties, future research should consider developing novel models of care in a scalable, cost-effective manner, for longer-term surveillance of children born preterm. Further, an increased understanding of neonatal and social risk factors underpinning the Preterm Behavioral Phenotype will guide targeted and preventative interventions.

## Data Availability

The raw data supporting the conclusions of this article will be made available by the authors, without undue reservation.
