# Supplementary material for: Risk profiles of the preterm behavioral phenotype in children aged 3 to 18 years
Source: Front Pediatr. 2023 Oct 19;11:1084970. doi: 10.3389/fped.2023.1084970 (PMC10620930; doi:10.3389/fped.2023.1084970)

Supplementary Table S1: Model fit indices of latent profiles allowing covariance between outcomes.

Number of Profiles	AIC	BIC	SABIC	Entropy	VLMRLRT	LMRALRT		LL	Smallest Class %
					p	Statistic	*p*		
1	17656	17708	17680	N/A	N/A	N/A	N/A	-8819.30	100
2	17138	17213	17172	.84	<.001	510.09	<.001	-8556.06	15
3	16953	17051	16997	.81	.02	186.94	.02	-8459.59	5
4	16812	16934	16867	.82	.06	143.70	.06	-8385.43	4
5	16693	16837	16758	.83	.05	123.84	.05	-8321.53	3
6	16629	16797	16705	.84	.18	69.15	.18	-8285.84	1
7	16564	16755	16650	.79	.05	70.71	.06	-8249.35	1
8	16521	16735	16618	.79	.10	49.28	.10	-8223.92	0.4
9	16502	16740	16609	.78	.59	26.04	.60	-8210.48	1
10	16472	16732	16589	.78	.58	18.43	.59	-8191.02	0.3

Note. AIC: Akaike Information Criterion; BIC: Bayesian Information Criterion; SABIC: Sample Size Adjusted Bayesian Information Criterion; VLMRLRT: Vuong-Lo-Mendell-Rubin Likelihood Ratio Test; LMRALRT: Lo-Mendell-Rubin-Adjusted Likelihood Ratio Test; LL: Log Likelihood; N/A: Not Applicable.

Supplementary Table S2: Probability of profile membership by sex, gestational age, and chronological age.

**Characteristics, Membership Probability (95% Confidence Interval)**	***n***	**Profile 1: Low Expression**	**Profile 2: Moderate Expression**	**Profile 3: High Expression**
**Sex**				
Female	1,127	.53 (.38–.67)	.35 (.22–.50)	.12 (.10 –.15)
Male	1,279	.71 (.65–.77)	.22 (.18–.29)	.07 (.04–.11)
**Gestational age**				
Moderate/late preterm, 32–<37 weeks	1,052	.75 (.71–.78)	.19 (.16–.23)	.06 (.04–.08)
Very preterm, 28–<32 weeks	785	.71 (.65–.76)	.22 (.18–.27)	.07 (.04–.10)
Extremely preterm, <28 weeks	569	.72 (.67–.76)	.24 (.20–.29)	.04 (.03–.07)
**Chronological age**				
Preschool, 3–5 years	1,123	.75 (.69–.80)	.19 (.14–.23)	.07 (.05–.09)
School-age, 6–9 years	748	.70 (.64–.74)	.25 (.21–.29)	.06 (.04–.09)
Early adolescence, 10–14 years	394	.68 (.61–.73)	.28 (.23–.35)	.04 (.02–.08)
Late adolescence, 15–18 years	141	.56 (.44–.66)	.23 (.14–.37)	.21 (.13–.33)

Supplementary Figure 1: Bar graph representation of the latent profile analysis for subdomain scores for ADHD, ASD, and anxiety.

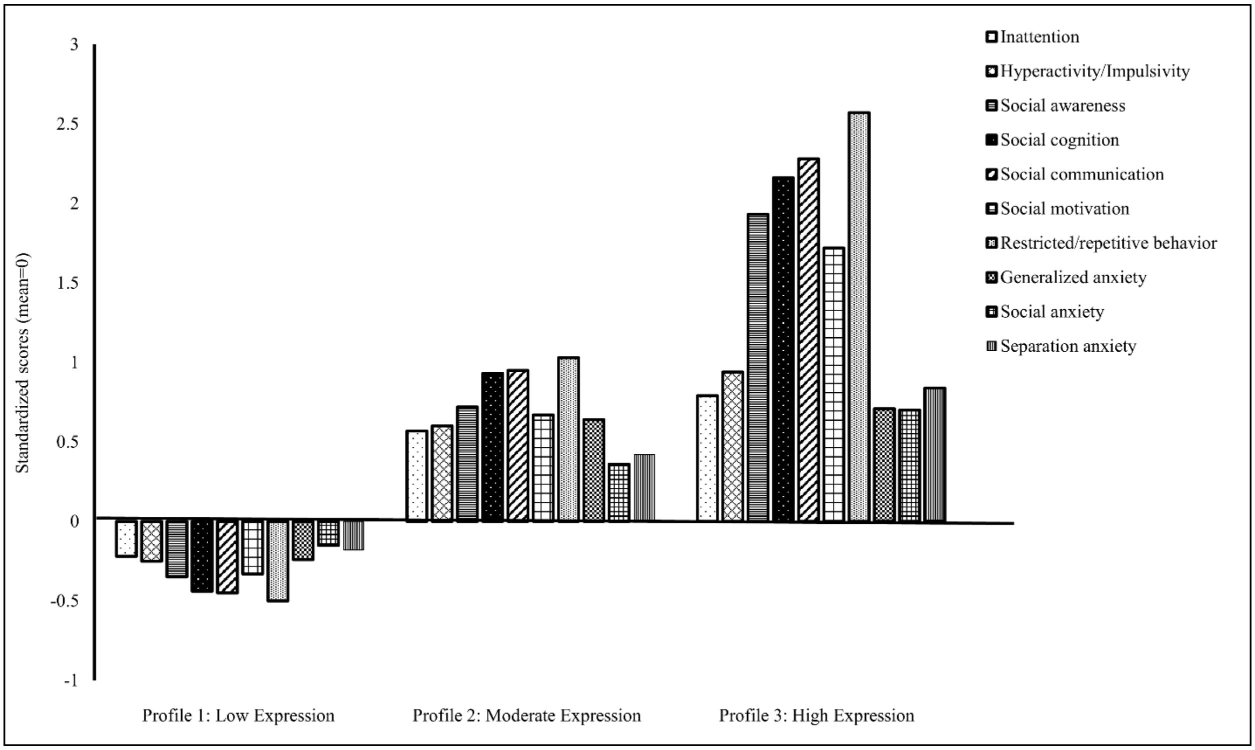

Supplement: Supplementary file 1 [file Datasheet1.pdf]
